# Trainability of Muscular Activity Level during Maximal Voluntary Co-Contraction: Comparison between Bodybuilders and Nonathletes

**DOI:** 10.1371/journal.pone.0079486

**Published:** 2013-11-15

**Authors:** Sumiaki Maeo, Takumi Takahashi, Yohei Takai, Hiroaki Kanehisa

**Affiliations:** 1 Graduate School of Physical Education, National Institute of Fitness and Sports in Kanoya, Kanoya, Japan; 2 Research Fellow of the Japan Society for the Promotion of Science, Tokyo, Japan; 3 Sports and Life Science, National Institute of Fitness and Sports in Kanoya, Kanoya, Japan; University of Alberta, Canada

## Abstract

Antagonistic muscle pairs cannot be fully activated simultaneously, even with maximal effort, under conditions of voluntary co-contraction, and their muscular activity levels are always below those during agonist contraction with maximal voluntary effort (MVE). Whether the muscular activity level during the task has trainability remains unclear. The present study examined this issue by comparing the muscular activity level during maximal voluntary co-contraction for highly experienced bodybuilders, who frequently perform voluntary co-contraction in their training programs, with that for untrained individuals (nonathletes). The electromyograms (EMGs) of biceps brachii and triceps brachii muscles during maximal voluntary co-contraction of elbow flexors and extensors were recorded in 11 male bodybuilders and 10 nonathletes, and normalized to the values obtained during the MVE of agonist contraction for each of the corresponding muscles (% EMG_MVE_). The involuntary coactivation level in antagonist muscle during the MVE of agonist contraction was also calculated. In both muscles, % EMG_MVE_ values during the co-contraction task for bodybuilders were significantly higher (*P*<0.01) than those for nonathletes (biceps brachii: 66±14% in bodybuilders vs. 46±13% in nonathletes, triceps brachii: 74±16% vs. 57±9%). There was a significant positive correlation between a length of bodybuilding experience and muscular activity level during the co-contraction task (r = 0.653, *P* = 0.03). Involuntary antagonist coactivation level during MVE of agonist contraction was not different between the two groups. The current result indicates that long-term participation in voluntary co-contraction training progressively enhances muscular activity during maximal voluntary co-contraction.

## Introduction

Simultaneous voluntary contractions of antagonistic pairs (co-contraction) produce resistive forces that act against each other to produce zero net torque [1]. It has been suggested that co-contraction can be a modality for improving the strength capability of exercised muscles [2], [3], and is used in both rehabilitation and strength training regimens [4]. In fact, a recent study [2] reported that a training program in which subjects performed voluntary co-contractions of elbow flexors and extensors produced significant increases in the strength capability of both muscle pairs without the use of an external load as resistance. On the other hand, it also appears that antagonistic muscle pairs cannot be fully activated simultaneously, even with maximal effort, under conditions of co-contraction [3], [5]. For example, the electromyogram (EMG) activities of elbow flexors and extensors during the co-contraction task performed at the elbow joint angle of 90–100 deg, expressed as the value relative to that during maximal voluntary effort (MVE) of agonist contraction (% EMG_MVE_), have been shown to be 40–60% and 60–75%, respectively [3], [5], [6].

In the prior study cited above [5], the measurement sessions were repeated three times to determine whether the results were reproducible despite additional practice because an attempt to maximize muscle activation voluntarily through co-contraction was not a task subjects initially approached with a clear sense of strategy. As a result, the % EMG_MVE_ values were similar with repetitive testing and were always below the maximum for both elbow flexors and extensors. In addition, a recent study [6] showed that a 4-wk of maximal voluntary co-contraction training performed on elbow flexors and extensors did not change the muscular activity levels during the task. These findings tempt us to speculate that muscular activation levels during voluntary co-contraction are always below those during MVE of agonist contraction, even after subjects are familiarized with the task. To our knowledge, however, no study has investigated whether muscular activity level during voluntary co-contraction is increased (trainable) after performing the task for a long period. To discuss the efficacy of voluntary co-contraction as a training modality for improving muscle function, this issue should be clarified.

De Luca and Mambrito [7] suggested that agonist-antagonist groups may be controlled by a common drive. Their model proposes that separate “flex” and “extend” commands exist to control reciprocal actions, whereas a third command, “coactivation” controls the pure co-contraction of both agonist and antagonist muscles [2], [7]. This notion of different controls for the different aspects of agonist-antagonist contraction states has been supported by electrophysiological evidence from other studies [8], [9]. It has also been shown that some cortical cells are active during co-contraction tasks, but not during flexion-extension movements [10]. Experiments using magnetic stimulation of the motor cortex have provided evidence for differential cortical control of flexion-extension movements and co-contractions [11], [12]. Thus, it is believed that the ability to co-contract can be improved after voluntary co-contraction training, owing to the increased neural drive to the third command (a common drive) during co-contraction [2], [13]. In addition, there is also a possibility that long-term participation in voluntary co-contraction training might reduce the influence of inhibitory system during co-contraction of antagonistic muscles. During maximal voluntary muscle contraction, efferent motor neuronal output is inhibited by central descending pathways, afferent inflow from group Ib Golgi organ afferents, group Ia and II muscle spindle afferents, group III muscle afferents, and by recurrent Renshaw inhibition [14]. However, all of these pathways may exhibit adaptive plasticity with training [14]. Taking these into account, therefore, it seems that any of these inhibitory systems occurring during the co-contraction task might be less involved following long-term co-contraction training as a result of chronic neural adaptations to the task, and consequently, the ability to co-contract antagonistic muscle pairs would be enhanced.

Given the competitive nature of bodybuilding, bodybuilders are frequently required to perform posing with static contractions [15], which is considered to be the same physical action as voluntary co-contraction [16]. Therefore, although there is no substantial evidence, it has been suggested that elite bodybuilders can control the activation levels of their individual muscles [15] and may be able to fully activate nearly every muscle by performing co-contraction [16]. Thus, clarifying the difference between elite bodybuilders and untrained individuals (nonathletes) in the EMG activities during maximal voluntary co-contraction will provide useful information for discussing whether muscular activity level during maximal voluntary co-contraction is trainable.

In the present study, we measured the muscular activation levels of elbow flexors and extensors during maximal voluntary co-contraction task in bodybuilders and nonathletes. We also quantified “involuntary” coactivation level of antagonist muscles during the MVE of agonist contraction to identify whether the activation strategy during MVE of agonist contraction is the same between bodybuilders and nonathletes, and whether the difference is specific to the “voluntary” co-contraction task. The purpose of the present study was to clarify whether muscular activity level during maximal voluntary co-contraction is trainable by comparing those performed by bodybuilders and nonathletes. We hypothesized that bodybuilders show higher muscular activity levels during maximal voluntary co-contraction than nonathletes as a result of neuromuscular adaptations to the repeated use of maximal voluntary co-contraction for a long period.

## Materials and Methods

### Subjects

Eleven male bodybuilders and ten nonathletes participated in this study. The experience of bodybuilding in the bodybuilders was 14.9±9.4 (mean ± SD) years. The means and SDs of age, body height, and body mass in the subjects were 40.8±11.0 yrs, 165.9±4.1 cm, and 69.0±5.7 kg for bodybuilders, and 22.0±1.6 yrs, 167.5±4.1 cm, and 64.6±6.6 kg for nonathletes, respectively. All bodybuilders were ranked at an elite level by their successful performance in domestic competitions. All nonathletes were habitually active, but none was involved in any type of exercise program (≥30 min/day, ≥2 days/week). This study was approved by the Ethics Committee of the National Institute of Fitness and Sports in Kanoya and was consistent with institutional ethical requirements for human experimentation in accordance with the Declaration of Helsinki. Prior to the measurement session, all subjects were fully informed about the procedures and possible risks involved as well as the purpose of the study, and their written informed consent was obtained.

### Procedure

In the measurement session, all subjects performed a static MVE task and a maximal voluntary co-contraction task of the elbow flexors and extensors. Firstly, the MVE tasks for elbow flexion and extension were performed for the purpose of normalization. In the MVE tasks, as well as subsequent co-contraction tasks, the surface EMG activities of the long head of each of the biceps brachii and triceps brachii muscles were recorded. The selection of the long head was based on the concept of a “muscle equivalent”, where one head of each of the biceps and triceps brachii muscles represents the whole muscle group, applies to both of the elbow flexors and extensors [17]–[19]. In fact, previous studies have shown that there is no difference in force-EMG relationship among heads of either biceps brachii or triceps brachii muscles [17]–[19]. Furthermore, in the recent study [6], there was no difference in % EMG_MVE_ values during the co-contraction task among heads of either biceps or triceps brachii muscles. On the basis of these aspects, we examined only the long head of each of the biceps brachii and triceps brachii muscles. In the MVE tasks, the participants developed force against manual resistance which was provided by an examiner. This is often used when normalizing EMG [16], [20], [21]. In this study, the examiner used both arms to provide resistance against the subjects’ one-arm elbow flexion/extension. This enabled the examiner to provide sufficient resistance against the subjects without joint movements while they performed the MVE tasks. The positions and tasks for MVE were adopted following the guidelines previously reported [3], [5], and each of the MVE tasks was performed as follows.

#### Elbow flexion

The subjects were seated in a chair with their right arm flexed at 90 deg of the elbow joint angle (upper arm vertical to the floor) and the forearm in a neutral position. The subjects then performed maximal static elbow flexion against manual resistance.

#### Elbow extension

In the same position as for the elbow flexion, the subjects performed maximal static elbow extension against manual resistance.

Prior to the MVE tasks, the subjects were asked to exert submaximal force statically against manual resistance at each of the test positions to familiarize themselves with the test procedure. After the completion of the process of warming-up and a rest period of 3 min, the subjects were encouraged to exert maximal force for 5 s two times with at least 3 min between trials to exclude the influence of fatigue. After the completion of the MVE tasks, the subjects performed a maximal voluntary co-contraction task for elbow flexors and extensors. The co-contraction task was performed as follows.

#### Co-contraction

In the same position as for the elbow flexion and extension, the subjects were instructed to perform simultaneous contraction of the elbow flexors and extensors with maximal effort for 5 s.

During the MVE and co-contraction tasks, the EMG biofeedback and verbal encouragement were provided [22]. The subjects were instructed not to perform any other actions such as shoulder abduction or forearm pronation/supination during the tasks. An examiner visually checked the subjects’ posture during the tasks to ensure that there was no joint movements. In every trial of the MVE and co-contraction tasks, we calculated the root-mean-square (RMS) amplitude of EMG with a procedure as described below and confirmed whether there was an apparent difference (more than 10%) between the two trials within the same task. On the basis of these criteria, if the researcher and/or subjects considered that the prior trial was unsuccessful, additional trial was performed. For most subjects in both bodybuilders and nonathletes, however, only two trials were performed for each task in the measurement sessions because they could complete the tasks without joint movements and an apparent difference in the RMS value between the trials. Thus, there was no significant difference in the number of trials performed by two groups.

### EMG Measurements and Analysis

**Figure 1:**
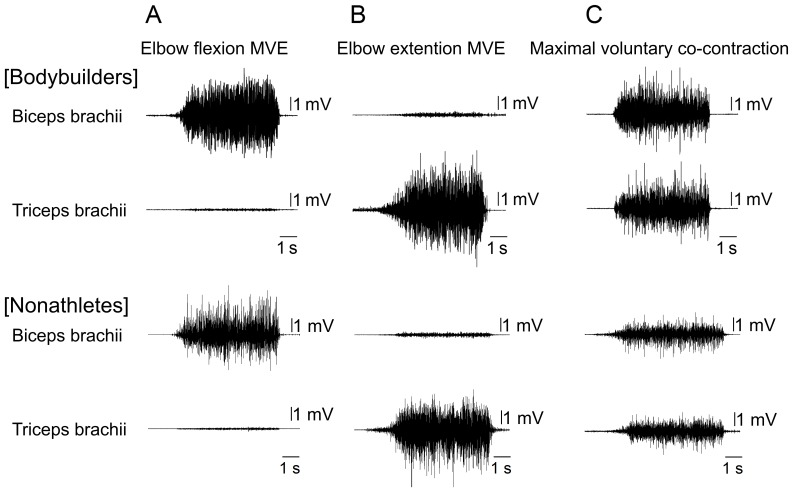
Example data. Example data of the EMGs of the biceps brachii (top row) and the triceps brachii (bottom row) during MVE tasks of elbow flexion (A) and elbow extension (B), and during maximal voluntary co-contraction task (C) for each of the bodybuilders and nonathletes.

In the static MVE and co-contraction tasks, the surface EMG activities of the long head of each of the biceps brachii and triceps brachii muscles of the right arm were measured by a bipolar configuration using a portable EMG recording apparatus (ME6000T16; MEGA Electronics, Finland). The guidelines for electrode locations described in previous reports [5], [6] were followed and a B-mode ultrasound apparatus (Prosound 2; Aloka, Japan) was used for detecting the directions of muscle fiber (fascicle) and positioning the electrodes over muscles because bodybuilders have been shown to have increased pennation angles [23]. Ag-AgCl electrodes of 15 mm diameter (N-00-S Blue sensor; Ambu, Denmark) were attached over the bellies of the muscles with an interelectrode distance of 20 mm after the skin surface had been shaved, rubbed with sandpaper, and cleaned with alcohol. Another electrode for each muscle was attached lateral to the recording electrodes and functioned as a ground electrode as well as a preamplifier. The EMG signals were 412-fold-amplified through the preamplifier, A/D-converted through a band-pass filter (8–500 Hz/3 dB) at a sampling frequency of 2,000 Hz, and stored on a personal computer. From EMG data, the RMS amplitude of EMG for each muscle was calculated using data analysis software (Chart version 7; ADInstruments, Australia). For biceps brachii and triceps brachii muscles, elbow flexion and elbow extension, respectively, were used for each of the MVE tasks. In the MVE and co-contraction tasks, the EMG data during the middle 3 s of maximal effort (5 s) were analyzed in each muscle and averaged across two trials, and EMGs of each muscle during co-contraction tasks are expressed as the value relative to those during MVE of each muscle (% EMG_MVE_) [3], [5]. In both groups, the RMS values during MVE and co-contraction tasks did not show significant differences between the 1^st^ and 2^nd^ trials. Averaged over the groups, the mean value of the relative difference in RMS value between the 1^st^ and 2^nd^ trials in MVE task was 4.8% for biceps brachii and 6.9% for the triceps brachii muscle. The corresponding value in co-contraction task was 6.2% for biceps brachii and 7.3% for the triceps brachii muscle. Similarly, from the EMG data of antagonist muscles during MVE tasks, the involuntary coactivation level of each muscle was determined over the same epoch and normalized as the value relative to that during the MVE trial in which those muscles act as agonists (% EMG_MVE_) [24]–[26] (Figure 1). The repeatability of % EMG_MVE_ measurements during the tasks was assessed on 2 separate days in a pilot study with 6 young adult men. In each of the prescribed tasks, there was no significant difference between the % EMG_MVE_ values of the two measurements. The intraclass correlation coefficients (ICC) and coefficient of variations (CV) for muscular activity levels during the co-contraction task were 0.883 and 7.1%, respectively, for biceps brachii muscle, and 0.811 and 10.2%, respectively, for triceps brachii muscle. Those values for involuntary coactivation level during MVE of agonist contraction tasks were 0.872 and 7.8%, respectively, for biceps brachii muscle, and 0.934 and 5.9%, respectively, for triceps brachii muscle.

### Statistical Analysis

Descriptive data are shown as means ± SDs. A two-way (2 groups×2 muscles) repeated measures ANOVA was used to test the effects of group and muscle and their interaction on muscular activity level during co-contraction task and involuntary coactivation level during MVE task. We also performed a simple linear regression analysis (Pearson’s correlation coefficient) to describe the relationship between a length of bodybuilding experience and % EMG_MVE_ during the co-contraction task. In this analysis, the average value of % EMG_MVE_ for the two muscle groups was calculated and used as a representative value of muscular activation level during the co-contraction task. Statistical significance was set at P<0.05. All data were analyzed using SPSS software (SPSS statistics 20; IBM, Japan).

## Results

**Figure 2:**
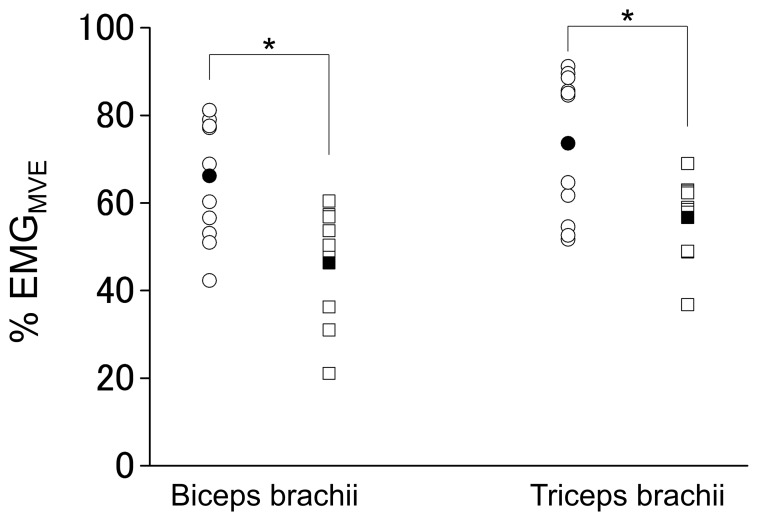
Muscular activation level during maximal voluntary co-contraction. Muscular activation level (% EMG_MVE_) during maximal voluntary co-contraction in bodybuilders (circle) and nonathletes (square). The % EMG_MVE_ values for both biceps brachii (bodybuilders: 66±14% vs. nonathletes: 46±13%) and triceps brachii muscles (74±16% vs. 57±9%) were significantly higher in bodybuilders than in nonathletes. Open and closed symbols indicate individual and mean values, respectively.

**Figure 3:**
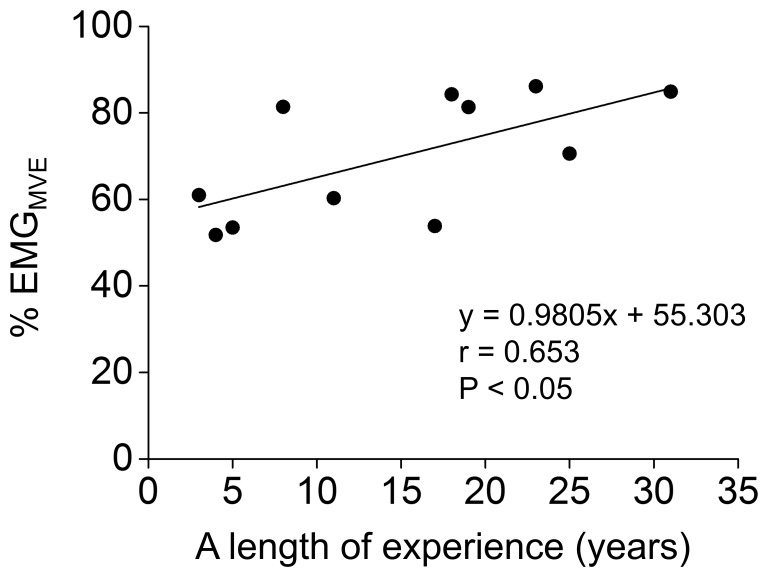
Relationship between bodybuilding experience and muscular activity level during maximal voluntary co-contraction task. Significant positive correlation was found between a length of bodybuilding experience and % EMG_MVE_ (averaged over biceps and triceps brachii muscles) during maximal voluntary co-contraction task.

**Figure 4:**
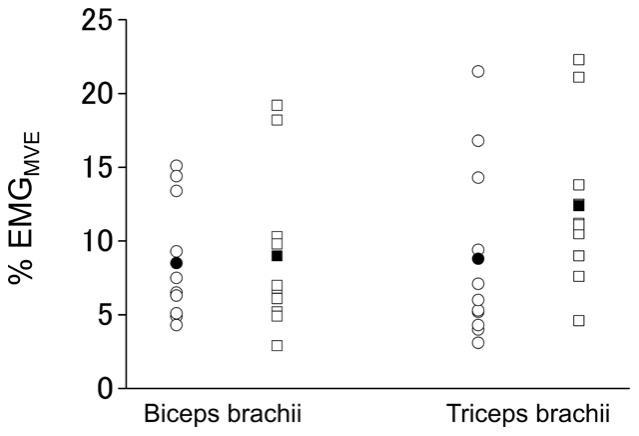
Involuntary antagonist coactivation level during MVE of agonist contraction. Involuntary antagonist coactivation level (% EMG_MVE_) during MVE tasks in bodybuilders (circle) and nonathletes (square). The % EMG_MVE_ values for both biceps brachii (during elbow extension MVE: 9±4% vs. 9±6%) and triceps brachii muscles (during elbow flexion MVE: 9±6% vs. 12±6%) were not different between groups. Open and closed symbols indicate individual and mean values, respectively.

% EMG_MVE_ values during the co-contraction task had a significant (P<0.01) main effect of group without a significant main effect of muscle or their interaction, indicating that the % EMG_MVE_ values for both biceps brachii and triceps brachii muscles were significantly higher in bodybuilders than in nonathletes (Figure 2). There was a significant positive correlation between a length of bodybuilding experience and muscular activity level during the task (r = 0.653, P = 0.03, Figure 3). There were no significant main effects of group and muscle or their interaction in involuntary coactivation level during MVE of agonist contraction (Figure 4).

## Discussion

As hypothesized at the start of this study, the % EMG_MVE_ values during maximal voluntary co-contraction task were significantly higher in bodybuilders than in nonathletes in the two muscle groups. This result supports the assertion that bodybuilders can activate their individual muscles during maximal voluntary co-contractions more than nonathletes. Furthermore, a significant positive correlation was found between a length of bodybuilding experience and muscular activity level during the co-contraction task. This suggests that muscular activity level during co-contraction can be progressively enhanced by continuing co-contraction training for a long period. Another finding obtained here was that involuntary coactivation levels during MVE of agonist contraction were similar between the two groups. It is known that resistance training changes not only agonist but also antagonist activities during force production, and their adaptations appear to be specific to the type of exercise used in training [27]–[29]. Thus, it might be assumed that while long-term participation in voluntary co-contraction training enhances muscular activity during maximal voluntary co-contraction, the contraction modality would also increase involuntary antagonist coactivation level during the MVE of agonists alone. However, the current results suggest that, in spite of the contraction modality, co-contraction training would not increase involuntary antagonist coactivation level during contractions of agonist alone, even if it is performed over years.

In the nonathletes, the % EMG_MVE_ values during voluntary co-contraction task were 46% for the biceps brachii and 57% for the triceps brachii muscles. These values are comparable to those reported in previous studies using untrained individuals [3], [6], in which the task was performed at the elbow joint angle of 90 deg and the observed values corresponding to % EMG_MVE_ were 40–60% for the biceps brachii and 60–66% for the triceps brachii muscles. Thus, it can be considered that the % EMG_MVE_ values observed in the nonathletes are representative values of those during maximal voluntary co-contractions of elbow flexors and extensors for untrained individuals. On the other hand, there is a possibility that the lower % EMG_MVE_ values during voluntary co-contraction task for the nonathletes would be due to that they are not familiarized with the task. As described earlier, however, the % EMG_MVE_ values during the co-contraction task has been shown to be unchanged following a short-term (4 weeks) co-contraction training [6]. This finding denies that the observed difference between the bodybuilders and nonathletes in the % EMG_MVE_ values during voluntary co-contraction task would be simply attributable to whether or not they are familiarized with the task.

The reasons why antagonistic muscles cannot be fully activated under conditions of voluntary co-contraction even with maximal effort are unknown. Pashler [30] indicated that, when two tasks are performed simultaneously, the performance of each is often impaired. This phenomenon is referred to as dual-task interference [30], and it often occurs even when performing relatively simple tasks, and especially when the task is unfamiliar. Considering this, it seems that the task requiring simultaneous contractions of multiple muscles induces a similar phenomenon to the dual-task interference, and so it might have resulted in the lower % EMG_MVE_ values during the co-contraction task. For the bodybuilders, however, the dual-task interference would be unlikely to be the main factor limiting muscular activation levels during the co-contraction task because they often perform co-contraction tasks through the practice of posing in their training program. Notably, the practice for elbow flexors and extensors is one of the most frequently performed parts in their posing training [15].

As another possible factor explaining the lower activation levels of antagonistic muscles during maximal voluntary co-contraction, the influences of inhibitory interneurons on the antagonist motoneurons of each muscle group might also be considered. When performing voluntary contractions and exerting force, the activation of antagonist muscles is inhibited through reciprocal Ia inhibition, enabling economical force production [12]. In the case of voluntary co-contraction, therefore, it is likely that the muscle activation generated in one muscle group would receive suppression from the opposite activating muscle group through Ia inhibitory interneurons, limiting the maximal activation of both antagonistic muscles [3], [31]. This reflex phenomenon is thought to serve to prevent large tangential muscle forces, thereby limiting joint surface compressive forces and offering protection to the joint [5]. Thus, it seems that muscular activation levels during voluntary co-contraction never reach the maximum.

It is, however, also true that reflexes are adaptable to particular motor tasks [13], [32], [33]. During the co-contraction of antagonistic ankle muscles, the presynaptic inhibition of Ia afferents is increased and, as a result, the soleus H-reflex size is depressed [13], [34]. In addition, recurrent inhibition from Renshaw cells activated by motor axon collaterals is increased during co-contraction [13], [35]. This likely occurs to ensure that reciprocal inhibition is maintained low (inhibition of the reciprocal inhibition) and thus facilitates the simultaneous activation of the antagonistic muscles [13], [31]. Indeed, ballet dancers, who frequently perform co-contraction of ankle antagonistic muscles in their practice, are better at controlling and co-contracting these muscles, and have smaller H-reflex amplitudes than other trained individuals [33]. Perez et al. [13] indicated that the repeated performance of tasks involving co-contraction may lead to prolonged changes in reflex and corticospinal excitability. As mentioned earlier, bodybuilders frequently perform posing [15], which induces a similar contraction mode as co-contraction [16], and activate their muscles as much as possible in their competitions and regular training [15]. In their training program, for example, it is advocated that “one should practice posing until one has established total control over each of the muscles involved [15]”. Considering this, there is a possibility that, for bodybuilders, the magnitude of reciprocal Ia inhibition involved during the co-contraction task would be less owing to neural adaptations to the co-contraction training for a long period, which might have contributed to produce the higher % EMG_MVE_ values during the co-contraction task.

## Conclusions

The current study revealed that the % EMG_MVE_ values during maximal voluntary co-contraction of elbow flexors and extensors were significantly higher in bodybuilders than in nonathletes. Also, % EMG_MVE_ during the co-contraction task was positively correlated to the length of bodybuilding experience. These results suggest that the muscular activity level during maximal voluntary co-contraction can be progressively enhanced by voluntary co-contraction training for a long period, probably due to the improved ability to activate targeted muscles.
